# HIV treatment outcomes among people who acquired HIV via injecting drug use in the Asia‐Pacific region: a longitudinal cohort study

**DOI:** 10.1002/jia2.25736

**Published:** 2021-05-21

**Authors:** Win Min Han, Awachana Jiamsakul, Nur Afiqah Mohd Salleh, Jun Yong Choi, Bui Vu Huy, Evy Yunihastuti, Cuong Duy Do, Tuti P Merati, Yasmin M Gani, Sasisopin Kiertiburanakul, Fujie Zhang, Yu‐Jiun Chan, Man‐Po Lee, Romanee Chaiwarith, Oon Tek Ng, Suwimon Khusuwan, Rossana Ditangco, Nagalingeswaran Kumarasamy, Shashikala Sangle, Jeremy Ross, Anchalee Avihingsanon, TREAT Asia HIV Observational Database (TAHOD) of the International Epidemiology Databases to Evaluate AIDS (IeDEA) Asia‐Pacific Group, PS Ly, V Khol, FJ Zhang, HX Zhao, N Han, MP Lee, PCK Li, W Lam, YT Chan, N Kumarasamy, C Ezhilarasi, S Pujari, K Joshi, S Gaikwad, A Chitalikar, S Sangle, V Mave, I Marbaniang, S Nimkar, TP Merati, DN Wirawan, F Yuliana, E Yunihastuti, A Widhani, S Maria, TH Karjadi, S Oka, T Nishijima, JY Choi, S Na, JM Kim, YM Gani, NB Rudi, I Azwa, A Kamarulzaman, SF Syed Omar, S Ponnampalavanar, R Ditangco, MK Pasayan, ML Mationg, YJ Chan, WW Ku, PC Wu, E Ke, OT Ng, PL Lim, LS Lee, D Liang, OT Ng, A Avihingsanon, S Gatechompol, P Phanuphak, C Phadungphon, S Kiertiburanakul, A Phuphuakrat, L Chumla, N Sanmeema, R Chaiwarith, T Sirisanthana, J Praparattanapan, K Nuket, S Khusuwan, P Kantipong, P Kambua, KV Nguyen, HV Bui, DTH Nguyen, DT Nguyen, CD Do, AV Ngo, LT Nguyen, AH Sohn, JL Ross, B Petersen, MG Law, A Jiamsakul, R Bijker, D Rupasinghe

**Affiliations:** ^1^ Kirby Institute UNSW Sydney Australia; ^2^ HIV‐NAT Thai Red Cross AIDS Research Centre Bangkok Thailand; ^3^ Department of Social and Preventive Medicine Faculty of Medicine University of Malaya Kuala Lumpur Malaysia; ^4^ Centre of Excellence for Research in AIDS (CERiA) University of Malaya Kuala Lumpur Malaysia; ^5^ Division of Infectious Diseases Department of Internal Medicine Yonsei University College of Medicine Seoul South Korea; ^6^ National Hospital for Tropical Diseases Hanoi Vietnam; ^7^ Faculty of Medicine Universitas Indonesia ‐ Dr. Cipto Mangunkusumo General Hospital Jakarta Indonesia; ^8^ Bach Mai Hospital Hanoi Vietnam; ^9^ Faculty of Medicine Udayana University & Sanglah Hospital Bali Indonesia; ^10^ Hospital Sungai Buloh Sungai Buloh Malaysia; ^11^ Faculty of Medicine Ramathibodi Hospital Mahidol University Bangkok Thailand; ^12^ Beijing Ditan Hospital Capital Medical University Beijing China; ^13^ Taipei Veterans General Hospital Taipei Taiwan; ^14^ Queen Elizabeth Hospital Hong Kong China; ^15^ Research Institute for Health Sciences Chiang Mai Thailand; ^16^ Tan Tock Seng Hospital Tan Tock Seng Singapore; ^17^ Chiangrai Prachanukroh Hospital Chiang Rai Thailand; ^18^ Research Institute for Tropical Medicine Muntinlupa City Philippines; ^19^ Chennai Antiviral Research and Treatment Clinical Research Site (CART CRS) VHS‐Infectious Diseases Medical Centre VHS Chennai India; ^20^ BJ Government Medical College and Sassoon General Hospital Pune India; ^21^ TREAT Asia amfAR ‐ The Foundation for AIDS Research Bangkok Thailand; ^22^ Tuberculosis Research Unit Faculty of Medicine Chulalongkorn University Bangkok Thailand

**Keywords:** people who inject drugs, treatment outcomes, CD4 recovery, viral suppression, tuberculosis, HIV/AIDS, Asia‐Pacific

## Abstract

**INTRODUCTION:**

Data on HIV treatment outcomes in people who inject drugs (PWID) in the Asia‐Pacific are sparse despite the high burden of drug use. We assessed immunological and virological responses, AIDS‐defining events and mortality among PWID receiving antiretroviral therapy (ART).

**METHODS:**

We investigated HIV treatment outcomes among people who acquired HIV via injecting drug use in the TREAT Asia HIV Observational Database (TAHOD) between January 2003 and March 2019. Trends in CD4 count and viral suppression (VS, HIV viral load <1000 copies/mL) were assessed. Factors associated with mean CD4 changes were analysed using repeated measures linear regression, and combined AIDS event and mortality were analysed using survival analysis.

**RESULTS:**

Of 622 PWID from 12 countries in the Asia‐Pacific, 93% were male and the median age at ART initiation was 31 years (IQR, 28 to 34). The median pre‐ART CD4 count was 71 cells/µL. CD4 counts increased over time, with a mean difference of 401 (95% CI, 372 to 457) cells/µL at year‐10 (n = 78). Higher follow‐up HIV viral load and pre‐ART CD4 counts were associated with smaller increases in CD4 counts. Among 361 PWID with ≥1 viral load after six months on ART, proportions with VS were 82%, 88% and 93% at 2‐, 5‐ and 10‐years following ART initiation. There were 52 new AIDS‐defining events and 50 deaths during 3347 person‐years of follow‐up (PYS) (incidence 3.05/100 PYS, 95% CI, 2.51 to 3.70). Previous AIDS or TB diagnosis, lower current CD4 count and adherence <95% were associated with combined new AIDS‐defining event and death.

**CONCLUSIONS:**

Despite improved outcomes over time, our findings highlight the need for rapid ART initiation and adherence support among PWID within Asian settings.

## 1 INTRODUCTION

Globally, injecting drug use is a major public health concern leading to substantial health burden and transmission risk of blood‐borne infections from injecting equipment sharing behaviours. In a multi‐stage systematic review, it was estimated that there were nearly 4 million people who inject drugs (PWID) reside within the east and southeast Asia, with a population prevalence of 0.25% (95% confidence interval [CI], 0.19 to 0.31) [1]. Among the PWID population in the region, HIV and hepatitis C infections occur in 15% and 50% respectively [1].

The HIV cascade of care is a useful framework to monitor people living with HIV (PLHIV) from the first point of diagnosis to the ultimate goal of achieving high levels of HIV RNA‐1 viral load suppression [2]. Between these steps, linkage to HIV care, and initiation and adherence to antiretroviral therapy (ART) constitute the overall framework of the HIV care cascade towards attaining successful treatment, in which HIV transmission rates are reduced and disease progression is stalled. Although combined antiretroviral therapy (ART) has benefited millions of PLHIV and has reduced HIV‐associated mortality, studies have shown that PWID with HIV are at risk for poorer ART adherence [3, 4] and poorer virological and immunological responses to ART compared to the non‐injecting individuals [5, 6, 7].

The causes of suboptimal treatment outcomes and mortalities are likely multifactorial, including adherence problem [8, 9], opportunistic infections (such as tuberculosis infection) [10, 11], other comorbid conditions (such as hepatitis C virus co‐infection) [1], lack of social support and stigmatization [12]. A number of studies have also found significant links between poor treatment outcomes among PWID and a range of risk factors embedded within the social and structural environments including low socioeconomic status, unstable housing and incarceration [12]. More importantly, environmental exposures and social barriers such as lack of facilities and support to access sterile injection equipment, barriers to access healthcare services, and the criminalization and stigmatization of drug use have a significant impact on the HIV disease progression in this hard‐to‐reach population [13].

Despite the need to identify and target key interventions, little is known regarding HIV‐related treatment outcomes among HIV‐positive PWID living in the Asia‐Pacific region. The few studies that previously evaluated treatment outcomes among PWID in the region were cross‐sectional and had a short duration of follow‐up [14, 15, 16, 17]. In this study, we capitalized on access to long‐term ART follow‐up data of TREAT Asia HIV Observational Database (TAHOD) and aimed to assess the immunological and virological responses, AIDS‐defining events, and mortality exclusively among PWID receiving ART in the Asia‐Pacific region using data from TAHOD.

## 2 MATERIALS AND METHODS

### 2.1 Study design and population

This study is a longitudinal analysis evaluating HIV treatment outcomes among HIV‐positive PWID who have started ART. PLHIV who reported injecting drug use at enrolment as their primary mode of HIV acquisition in the TAHOD cohort between January 2003 and March 2019 were included. TAHOD is a multi‐site observational cohort study which includes 21 participating sites from 12 countries in the Asia‐Pacific region. Details of the cohort and its methods have been reported in previous studies [18, 19, 20]. Briefly, since 2003, TAHOD selectively enrolled PLHIV aged ≥18 years or older [18], who were more likely to remain in care. Patients were followed according to the local standard of care. As of March 2019, the TAHOD cohort included just over 9800 participants with an average follow‐up frequency of 3.83 per 1 person‐year and a median follow‐up time of 7.8 years (interquartile range [IQR], 4.6 to 11.2). TAHOD is entirely observational, and any tests or interventions are performed according to the site’s local practices.

### 2.2 Study outcomes and covariates

The first part of the study was to evaluate immunological and virological responses up to 10 years after ART initiation. Immunological and virological responses were measured by CD4 changes and the proportion of individuals with HIV viral suppression after ART initiation respectively. To measure immunological changes, we included patients with available baseline CD4 counts, defined as the latest pre‐ART CD4 count within six months of treatment initiation. Viral suppression (VS) was defined as HIV‐1 RNA <1000 copies/mL. As a sensitivity analysis, the threshold of 400 copies/mL was used to define VS.

The second part of the study was to explore a combined endpoint of AIDS‐defining events and all‐cause mortality and incidence of TB. AIDS‐defining events were defined as having a condition from the modified CDC AIDS‐indicator list, including tuberculosis (TB) or an AIDS‐related cancer. All‐cause mortality was defined as AIDS‐ and non‐AIDS‐related deaths. Causes of death were based on the review of the standardized Cause of Death (CoDe) form outlined by the D:A:D study [21]. Participants who had an AIDS event and died were counted as dead due to AIDS event. TB was defined as having either presumptive or definitive pulmonary, or extrapulmonary TB disease.

We considered a range of sociodemographic variables, including age (≤30, 31 to 40, 41 to 50 and ≥51 years), sex, and country income level (low‐middle and high income). Self‐reported adherence was collected during follow‐up visits from the visual analogue scale [3]. Additionally, treatment and clinical‐related variables were considered and modelled as time‐fixed covariates, including hepatitis B and C co‐infection, prior AIDS diagnosis or TB diagnosis preceding ART initiation, year of treatment initiation (≤ 2002, 2003 to 2007, 2008 to 2012 and 2013 to 2018). Hepatitis B (HBV) and C (HCV) co‐infections were defined as having an ever‐positive result of hepatitis B surface antigen and HCV antibody respectively. Time‐varying covariates included in the study were age, CD4 count, HIV‐1 RNA level, ART regimen (Non‐nucleoside reverse transcriptase inhibitor (NNRTI)‐based, and protease inhibitor (PI)‐based), body mass index (BMI) and self‐reported ART adherence (≤95% vs. >95%). Loss‐to‐follow‐up (LTFU) was defined as having no visit for at least 12 months prior to the data transfer date.

### 2.3 Statistical analysis

Participants with at least one CD4 measurement after six months on ART were included in the CD4 outcome analysis. Factors associated with CD4 changes up to 10 years from ART initiation were evaluated using repeated measure linear regression (generalized estimating equation (GEE) with exchangeable correlation matrix using robust covariance estimation), adjusting for site and time on ART. The proportion of patients with VL suppression was analysed descriptively. Participants with at least one viral load measurement after six months on ART were included.

Risk factors for TB, and combined AIDS‐defining event and death were evaluated by Cox proportional hazard models. Crude incidence rates of combined AIDS/death and new TB events were reported per 100 persons years (/100 PYS). TB incident cases were analysed as a separate outcome and also included in the AIDS events/death analysis.

Survival time was left truncated at ART initiation or cohort entry, whichever occurred last, and ended on the outcome of interest. Patients who did not experience the outcome were lost to follow‐up, or transferred out were censored on their last visit date. Covariates from the univariable analysis with *p* < 0.10 were fitted in the multivariable models using a backward stepwise selection process. Covariates with *p* < 0.05 in the multivariable models were considered significant. Given the potential disparities in healthcare systems across the sites, analyses were stratified by site. As a sensitivity analysis, factors associated with CD4 changes over time were also analysed using linear mixed effect models with a random intercept on patients. All analyses were performed using SAS software version 9.4 (SAS Institute Inc., Cary, NC, USA) and STATA software version 14.2 (STATA Corp., College Station, TX, USA).

### 2.4 Ethical considerations

Ethics approvals were obtained from respective local ethics committees of all TAHOD‐participating sites, the Kirby Institute (data management and statistical analysis centre) and TREAT Asia/amfAR (coordinating centre). Informed consent was waived unless specified by the local institutional review board at each participating site.

## 3 RESULTS

### 3.1 Participant characteristics

**Figure 1:**
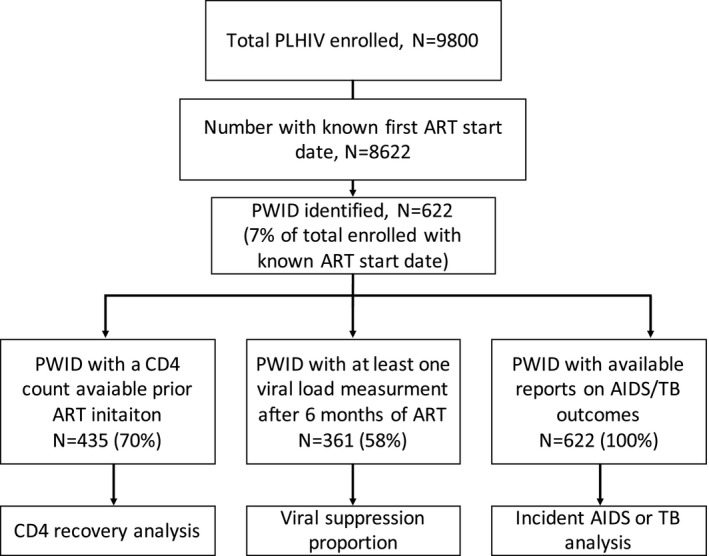
Participant inclusion flowchart.

Among 8622 participants with known first ART start date enrolled in the cohort until March 2019, 622 PWID (7.2%) were identified (Figure 1), of which 578 (93%) were male, and 510 (82%) were from low‐middle income countries. The participants included were from China (n = 9), Hong Kong SAR (n = 6), India (n = 2), Indonesia (n = 316), Malaysia (n = 51), the Philippines (n = 1), Singapore (n = 2), Taiwan (n = 9), Thailand (n = 35) and Vietnam (n = 191). The median age at ART initiation was 31 years (IQR, 28 to 34). The prevalence of HBV and HCV co‐infection was 10% and 71% respectively. Among PWID participants, 52 (8%) were co‐infected with both HBV and HCV. Over half (54%) had a history of an AIDS diagnosis, and over one‐third (34%) had been diagnosed with TB. Most (97%) started HIV treatment with non‐nucleoside reverse transcriptase inhibitor (NNRTI)‐based ART regimen, and the majority (59%) started ART in the 2005 to 2010 period. The median follow‐up time of our study group was 7.7 years (IQR, 3.8 to 10) with an average follow‐up rate of 4.89 (95% CI, 4.82 to 4.97) per 1 person‐year. Table 1 describes the characteristics of PWID participants in greater detail.

**Table 1:** Characteristics of PWID participants

	All PWID	AIDS/death events		Tuberculosis	
	Total N = 622 (100%)	Yes N = 102 (16.4%)	No N = 520 (83.6%)	Yes N = 35 (5.6%)	No N = 587 (94.4%)
Sex					
Male	578 (92.9%)	96 (94.1%)	482 (92.7%)	33 (94.3%)	545 (92.8%)
Female	44 (7.1%)	6 (5.9%)	38 (7.3%)	2 (5.7%)	42 (7.2%)
Age at ART initiation					
≤30	303 (48.7%)	46 (45.1%)	257 (49.4%)	12 (34.3%)	291 (49.6%)
31 to 40	271 (43.6%)	39 (38.2%)	232 (44.6%)	20 (57.1%)	251 (42.8%)
41 to 50	44 (7.1%)	16 (15.7%)	28 (5.4%)	3 (8.6%)	41 (7%)
51+	4 (0.6%)	1 (1%)	3 (0.6%)	0 (0%)	4 (0.8%)
Initial ART regimen					
NRTI+NNRTI	603 (97%)	97 (95.1%)	506 (97.3%)	34 (97%)	569 (96.9%)
NRTI+PI	19 (3%)	5 (4.9%)	14 (2.7%)	1 (3%)	18 (3.1%)
HBV coinfection					
Negative	434 (69.8%)	69 (67.6%)	365 (70.2%)	23 (65.7%)	411 (70%)
Positive	64 (10.3%)	11 (10.8%)	53 (10.2%)	3 (8.6%)	61 (10%)
Not tested	124 (19.9%)	22 (21.6%)	102 (19.6%)	9 (25.7%)	115 (20%)
HCV coinfection					
Negative	81 (13%)	12 (11.8%)	69 (13.3%)	4 (11.4%)	77 (13.1%)
Positive	440 (70.7%)	69 (67.7%)	371 (71.3%)	22 (62.9%)	418 (71.2%)
Not tested	101 (16.2%)	21 (20.6%)	80 (15.4%)	9 (25.7%)	92 (15.7%)
History of AIDS diagnosis					
No	287 (46%)	28 (27.4%)	259 (49.8%)	10 (28.6%)	397 (67.6%)
Yes	335 (54%)	74 (72.6%)	261 (50.2%)	25 (71.4%)	190 (32.4%)
History of tuberculosis					
No	413 (66.4%)	56 (54.9%)	357 (68.6%)	16 (45.7%)	357 (68.7%)
Yes	209 (33.6%)	46 (45.1%)	163 (31.4%)	19 (54.3%)	163 (31.3%)
Ever smoke					
No	45 (7.2%)	9 (8.8%)	36 (7%)	2 (5.7%)	43 (7.3%)
Yes	420 (67.5%)	55 (53.9%)	365 (70%)	24 (68.6%)	396 (67.5%)
Not reported	157 (25.2%)	38 (37.2%)	119 (23%)	9 (25.7%)	148 (25.2%)
Year of ART initiation					
<2005	97 (15.6%)	27 (26.3%)	70 (13.5%)	11 (31.4%)	86 (14.6%)
2005 to 2010	366 (58.8%)	55 (53.9%)	311 (59.8%)	21 (60.0%)	345 (58.8%)
>2010	159 (25.6%)	20 (19.6%)	139 (26.7%)	3 (8.6%)	156 (26.6%)
Country income level					
Low‐middle income countries	510 (82%)	77 (75.5%)	433 (83.3%)	25 (71.4%)	485 (82.6%)
High income countries	112 (18%)	25 (24.5%)	87 (16.7%)	10 (28.6%)	102 (17.4%)
Pre‐ART CD4+ count (cells/µL)					
≤50	199 (32%)	43 (42.2%)	156 (30%)	10 (28.6%)	189 (32.2%)
51 to 100	79 (12.7%)	13 (12.7%)	66 (12.7%)	2 (5.7%)	77 (13.1%)
101 to 200	100 (16.1%)	14 (13.7%)	86 (16.5%)	6 (17.1%)	94 (16%)
>200	80 (12.9%)	10 (9.8%)	70 (13.5%)	4 (11.4%)	76 (13%)
Not done	164 (26.3%)	22 (21.6%)	142 (27.3%)	13 (37.1%)	151 (25.7%)

ART, antiretroviral therapy; ART, antiretroviral therapy; NRTI, nucleoside reverse transcriptase inhibitor; NNRTI, non‐nucleoside reverse transcriptase inhibitor; PI, protease inhibitor.

### 3.2 Immunological and virological responses after ART initiation over time

**Figure 2:**
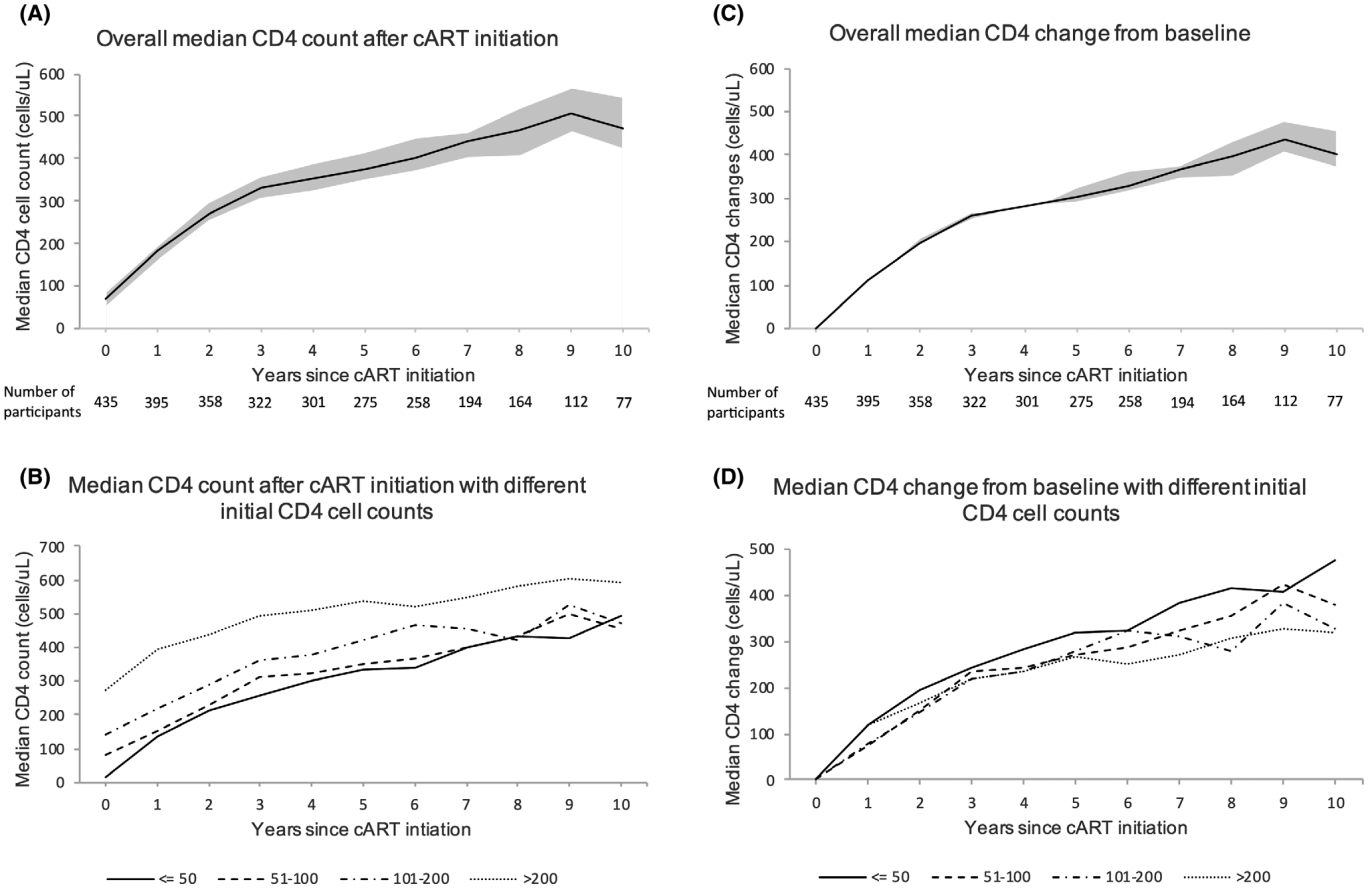
Longitudinal CD4 changes after ART initiation. A total of 435 people who inject drugs (PWID) contributed to pre‐ART CD4 count at baseline. Among them, 186 PWID had CD4 count ≤50 cells/µL, 77 had CD4 count 51 to 100 cells/µL, 96 had CD4 count 101 to 200 cells/µL and 76 had CD4 count >200 cells/µL at baseline respectively. **(A** and **C)** show overall median (95% CI) absolute CD4 count and median (95% CI) CD4 change from baseline respectively. **(B** and **D)** show median absolute CD4 count and median CD4 change from baseline with different pre‐ART CD4 counts (≤50, 51 to 100, 101 to 200 and >200 cells/µL).

The median pre‐ART CD4 was 72 (IQR, 55 to 88) cells/µL among 435 PWID (70%) who had a CD4 count available before ART initiation. An overall increase in CD4 was observed up to 10 years after ART initiation (Figure 2A). Among 76 PWID who were followed up at 10 years after ART initiation and had CD4 results available, there was a mean CD4 increase of 401 cells/uL (95% CI, 372 to 457) at the 10^th^ year of follow‐up (Figure 2C). Figure 2B and D show the median CD4 count and median CD4 change by initial CD4 count (≤50, 51 to 100, 101 to 200 and >200 cells/µL). Although PWID with different baseline CD4 counts showed increasing trends of CD4 during their follow‐up, only those with baseline CD4 >200 had achieved CD4 recovery of >500 since ART initiation.

Characteristics associated with mean CD4 changes over time in the multivariate analysis were current HIV viral load 401 to 100,000 copies/mL (difference = −65.3 cells/µL, 95% CI, −106.8 to −23.8, *p* = 0.002) and >100,000 copies/mL (−120.7, 95% CI, −174.8 to −66.7, *p* < 0.001) compared with viral load ≤400 copies/mL, pre‐ART CD4 51 to 100 cells/µL (−45.6, 95% CI, −79.8 to −11.4, *p* = 0.01), 101 to 200 cells/µL (−69.3 cells/µL, 95% CI, −103.7 to −34.8, *p* < 0.001) and >200 cells/µL (−97.1, 95% CI, −145.4 to −48.8, *p* < 0.001) compared with CD4 ≤50 cells/µL. The adherence variable was not included in the analysis of CD4 changes due to multicollinearity with HIV viral load (variance inflation factor, VIF = 14.3). We did not find an association with past AIDS or TB diagnosis, age at ART initiation, year of ART initiation, initial ART regimen and time‐updated BMI (Table 2). In the sensitivity analysis where we applied a linear mixed effect model with random effects on patients (Table S2), the estimates of the effects were similar to the estimates from the GEE model with an exchangeable correlation matrix.

**Table 2:** Factors associated with CD4 changes up to10 years after ART initiation

Total 435 PWID participants	Univariable			Multivariable		
	Difference (cells/µL)	95% CI	*p*‐value	Difference (cells/µL)	95% CI	*p*‐value
Time‐varying age (years)^a^						
≤30	Ref			Ref		
>30	−8.5	−31.5, 14.6	0.473	−7.5	−30.2, 17.6	0.510
Sex						
Male	Ref		0.736			
Female	−11.5	−78.4, 55.4	0.168			
Year of ART initiation						
<2005	Ref					
2005 to 2010	31.0	−27.4, 89.4	0.297			
>2010	−18.7	−80.4, 43.1	0.554			
HIV viral load (copies/mL)^a^						
≤400	Ref			Ref		**<0.001**
401 to 100,000	−92.5	−129.4, −55.6	<0.001	−65.3	−106.6, −23.9	**0.002**
>100,000	−157.7	−223, −92.3	<0.001	**−121.4**	**−176.0, −66.7**	**<0.001**
Not reported						
Pre‐ART CD4 (cells/µL)						
≤50	Ref		<0.001	Ref		**<0.001**
51 to 100	−46.6	−88.4, −4.8	0.029	**−45.3**	−79.5, −11.2	**0.009**
101 to 200	−60	−98.7, −21.3	0.002	**−69.5**	−104.0, −34.9	**<0.001**
>200	−89.5	−132.8, −46.2	<0.001	**−96.9**	−145.1, −48.7	**<0.001**
BMI (kg/m^2^)^a^						
<25			0.111			
≥25	33.4	−11.8, 78.5	0.147			
Not reported	−7.7	−23, 7.6	0.322			
Adherence^a^						
≤95%	Ref					
>95%	105.3	53.7, 156.9	<0.001			
Not reported						
ART regimen^a^						
No treatment	−11.2	−47.5, 182.5	0.554			
NRTI + NNRTI	Ref					
NRTI + PI	81.5	−17.8, 201.2	0.185			
Other combinations	22.5	−83.5, 201.2	0.365			
Ever smoke						
No	Ref					
Yes	5.5	−51.8, 62.9	0.850			
Not reported						
Hepatitis B co‐infection						
Negative	Ref					
Positive	−17.5	−67.6, 32.6	0.493			
Not tested						
Hepatitis C co‐infection						
Negative	Ref					
Positive	−33.1	−80.5, 14.2	0.170			
Not tested						
History of AIDS diagnosis						
No	Ref					
Yes	8.2	−23.1 to 39.5	0.608			
History of tuberculosis						
No	Ref					
Yes	4.2	−31.1, 39.6	0.814			

All model covariates were stratified by site. Global *p*‐values for age and CD4 are tests for trend. All other global *p*‐values are tests for heterogeneity excluding missing values. Time from ART initiation was also adjusted in the final model. *p*‐values in bold represent significant covariates in the final model. ART, antiretroviral therapy; NRTI, nucleotide reverse transcriptase inhibitor; NNRTI, non‐nucleotide reverse transcriptase inhibitor; PI, protease inhibitor; BMI, body mass index.

^a^ Age, HIV viral load, BMI, ART regimen and adherence data are time‐varying variables.

Among 361 PWID with at least one viral load measurement after six months on ART, the proportion with VS changed from 83% (120/145) in the first year of ART initiation, to 82% (115/140), 88% (82/93) and 93% (63/68) at 3, 5 and 10 years after starting of ART. In a sensitivity analysis when the threshold was used as 400 copies/mL, the proportion of VS remained the same as 1000 copies/mL. The proportion of VS over different years of ART initiation was comparable: 85%, 87% and 88% during years of <2005, 2005 to 2010 and >2010 respectively. In addition, 12 of the 25 PWID who did not have HIV VS at year 1 (48%) had died or LTFU by year 10, compared to 47 of 115 PWID who had VS (41%).

### 3.3 Incidence of AIDS/death events and TB among PWID

**Figure 3:**
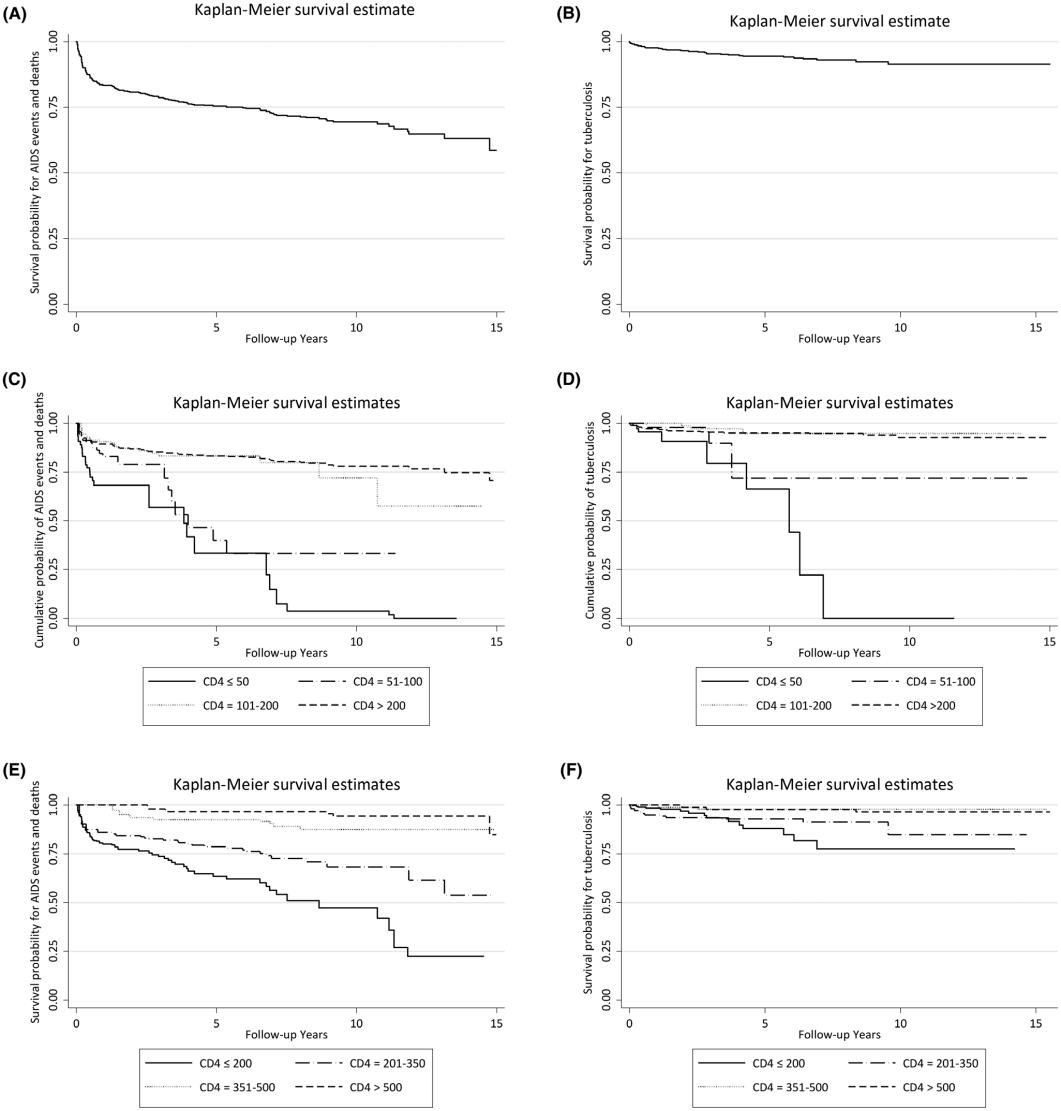
Kaplan–Meier curves for AIDS‐defining events/deaths tuberculosis among PWID. A total of 622 people who inject drugs (PWID) in the study analysis were recruited from 22 sites in Cambodia, China and Hong Kong SAR, India, Indonesia, Japan, Malaysia, the Philippines, Singapore, South Korea, Taiwan, Thailand and Vietnam. Site‐stratified survival graphs for **(A)** overall combined AIDS‐defining events and deaths, **(B)** overall tuberculosis, **(C)** combined AIDS‐defining events and deaths and **(D)** tuberculosis by time updated CD4 counts: ≤50, 51 to 100, 101 to 200 and >200 cells/µL, **(E)** combined AIDS‐defining events and deaths and F) tuberculosis by time‐varying CD4 counts: ≤200, 201 to 350, 351 to 500 and >500 cells/µL.

During 3347 PYS of follow‐up, there were 52 AIDS events and 50 deaths with a combined incidence rate of 3.05 (95% CI, 2.51 to 3.70)/100 PYS (Figure 3). Twenty‐five PWID died due to AIDS‐related events, with 17 deaths occurred due to non‐AIDS events and 8 of unknown causes. The median follow‐up time from six months after ART to the date of AIDS‐event or death was 4.5 years (IQR 2.4 to 7). Nearly 30% of non‐AIDS mortality were due to liver‐related mortality. In the multivariate analysis, previous AIDS diagnosis (HR = 1.86, 95% CI, 1.16 to 3.01, *p* = 0.01), current CD4 ≤50 cells/µL (HR = 5.79, 95% CI 2.99 to 11.25, *p* < 0.001) and CD4 of 51 to 100 cells/µL (HR = 3.39, 95% CI 1.59 to 7.19, *p* = 0.001) and ART adherence of ≤95% (HR = 11.1, 95% CI, 3.22 to 37.73, *p* < 0.001) were associated with incidence of new AIDS event or death (Table 3).

**Table 3:** Incidence rate of AIDS/deaths and associated factors among PWID

	Number of PWID	Follow‐up years	Number of AIDS/Deaths	Incidence rate (/100 pys)	95% CI	Univariable			Multivariable		
						HR	95% CI	*p*‐value	HR	95% CI	*p*‐value
Total	622	3347.73	102	3.05	2.51 to 3.70						
Sex											
Male	578	3105.48	96	3.09	2.53 to 3.78	Ref					
Female	44	242.25	6	2.48	1.11 to 5.51	0.67	0.29 to 1.56	0.356			
Time‐varying age (years)^a^											
≤30	–	496.72	21	4.23	2.76 to 6.48	Ref		0.121	Ref		
>30	–	2825.36	81	2.87	2.31 to 3.56	1.53	0.89 to 2.61	0.605	1.53	0.88 to 2.66	0.128
ART regimen^a^											
No treatment	–	28.87	3	10.39	3.35 to 32.22	2.07	0.57 to 7.51	0.267			
NRTI+NNRTI	–	2940.12	85	2.89	2.34 to 3.58	Ref					
NRTI+PI	–	327.03	10	3.06	1.65 to 5.68	1.09	0.50 to 2.39	0.816			
Others	–	46.44	4	8.61	3.23 to 22.95	1.74	0.46 to 6.61	0.417			
HBV co‐infection											
Negative	434	2376.97	69	2.9	2.29 to 3.68	Ref					
Positive	64	353.38	11	3.11	1.72 to 5.62	1.06	0.54 to 2.06	0.861			
Not tested	124	617.39	12	3.56	2.35 to 5.41						
HCV co‐infection											
Negative	81	438.18	12	2.74	1.56 to 4.82	Ref					
Positive	440	2436.53	69	2.83	2.24 to 3.59	1.18	0.60 to 2.31	0.631			
Not tested	101	473.03	21	4.44	2.89 to 6.81						
History of AIDS diagnosis											
No	287	1582.52	28	1.77	1.22 to 2.56	Ref			Ref		
Yes	335	1765.21	74	4.19	3.34 to 5.26	2.13	1.34 to 3.37	0.001	1.86	1.16 to 3.01	**0.010**
Not reported	300	1169.83	66	5.64	4.43 to 7.18						
BMI (kg/m^2^)^a^											
<25	–	2538.23	77	3.03	2.43 to 3.79	Ref					
≥25	–	438.21	9	2.05	1.07 to 3.95	0.59	0.28 to 1.22	0.158			
Not reported	–	371.29	16	4.31	2.64 to 7.03						
Year of ART initiation											
<2005	97	677.2	27	3.99	2.73 to 5.81	Ref		0.486			
2005 to 2010	366	1939.5	55	2.84	2.18 to 3.69	0.64	0.30 to 1.34	0.236			
>2010	159	705.4	20	2.84	1.83 to 4.39	0.68	0.31 to 1.57	0.371			
Adherence^a^											
≤95%	–	69.26	5	7.22	3.00 to 17.34	17.27	5.12 to 58.24	<0.001	11.1	3.22 to 37.73	**<0.001**
>95%	–	1634.32	14	0.86	0.51 to 1.45	Ref			Ref		
Not reported	–	1644.15	83	5.05	4.07 to 6.26			<0.001			**<0.001**
CD4+ cell count (cells/µL)^a^											
≤50	–	88.43	28	31.66	21.86 to 45.86	8.56	4.49 to 16.35	<0.001	5.79	2.99 to 11.25	**<0.001**
51 to 100	–	110.30	15	13.60	8.20 to 22.56	5.08	2.49 to 10.37	<0.001	3.39	1.59 to 7.19	**0.001**
101 to 200	–	430.36	15	3.49	2.10 to 5.78	1.92	0.99 to 3.71	0.052	1.54	0.79 to 3.00	0.206
>200	–	2534.47	39	1.54	1.12 to 2.11	Ref			Ref		
Not done	–	184.17	5	2.71	1.13 to 6.52						
HIV viral load (copies/mL)^a^											
≤400	–	1736.97	29	1.67	1.16 to 2.40	Ref		0.02			
401 to 100000	–	324.71	17	5.24	3.25 to 8.42	2.3	1.15 to 4.6	0.019			
>100000	–	117.65	22	10.2	5.79 to 17.96	3.1	1.33 to 7.23	0.009			
Not done	–	1168.41	44	3.77	2.80 to 5.06						

*p*‐values in bold represent significant covariates in the final model. Global *p*‐values are test for heterogeneity excluding missing values. Not reported/not done categories were included in the multivariable analysis, but not displayed in the table.

Abbreviations: ART, antiretroviral therapy; BMI, body mass index; NNRTI, non‐nucleotide reverse transcriptase inhibitor; NRTI, nucleotide reverse transcriptase inhibitor; PI, protease inhibitor.

^a^ Age, CD4, HIV viral load, adherence and BMI are time‐varying variables.

There were 35 new TB cases among 622 PWID. The incidence rate for TB was 1.01 (95% CI, 0.73 to 1.41)/100 PYS. Past TB diagnosis (HR = 2.69, 95% CI 1.23 to 5.87, *p* = 0.013), current age (>30 years, HR = 3.40, 95% CI, 1.24 to 9.34, *p* = 0.017; vs. ≤30 years), ART adherence ≤95% (HR = 24.23, 95% CI 1.62 to 42.19, *p* = 0.013) and current CD4 (≤50 cells/µL, HR = 5.53, 95% CI 2.05 to 14.97, *p* = 0.001; vs. >200 cells/µL) were associated with TB diagnosis (Table S1).

## 4 DISCUSSION

This study evaluated longitudinal immunological and virological outcomes and incidence of AIDS/death and TB among the TAHOD PWID population. CD4 counts and proportions of viral suppression continued to increase up to 10 years post‐ART initiation. Lower pre‐ART CD4 and lower current HIV viral load were associated with greater CD4 increase over the years of follow‐up. There were high proportions of HBV and HCV co‐infections among the population. In addition, previous AIDS diagnosis, lower current CD4 counts and suboptimal treatment adherence were associated with having an AIDS event/mortality in our study group.

Our study found that PWID with lower pre‐ART CD4 experienced greater CD4 gains after starting ART, compared to those with higher pre‐ART CD4. The overall rate of change was faster in the first three years after starting ART. This is consistent with previous studies which showed a greater CD4 changes in the early years of treatment initiation [22, 23]. Furthermore, only those with pre‐ART CD4 >200 cells/µL had their CD4 increase to above 500 cells/µL during follow‐up. Previous reports also suggested that individuals living with HIV who had higher CD4 at ART initiation predicted better CD4 recovery over the course of treatment [24, 25]. Findings from our study highlight the importance of addressing PWID presenting late to care in order to maximize their treatment response over time [26].

Current HIV viral load was associated with CD4 changes following ART initiation among PWID. Individuals with HIV VL ≤400 copies/mL during follow‐up had greater CD4 gain over time, compared to those who had a higher viral load. Higher viral load after ART initiation may be a result of treatment interruption or non‐adherence, which could be caused by a range of factors including ongoing injecting drug use, incarceration periods or poor engagement in opioid agonist therapy [27]. Individuals who started treatment during earlier years may have started with less effective ART regimens which potentially lead to slower viral suppression rate and indirectly affected CD4 changes over time. However, we found calendar years of treatment initiation were not statistically associated with CD4 changes over time.

There was a high proportion of VS in the cohort over the follow‐up period and most of our PWID in the study have started with NNRTI‐based ART regimen. However, the proportion of VS did not differ significantly according to year of ART initiation, which was categorized according to time periods when treatment guidelines would have been changing. Despite this, the high proportion of VS during follow‐up suggests PWID have responded well to HIV treatment. This highlights the importance of having PWID linked to care immediately after HIV diagnosis under the “Treat All” strategies for the implications of “Treatment‐as‐Prevention” as well as to optimize treatment outcomes among the population.

There was also a high prevalence of HBV and HCV co‐infections among our PWID participants. Nearly 30% of deaths were due to liver‐related problems. The burden of three blood‐borne infections (HIV, HBV, and HCV infections) among the PWID population was estimated to have enormous health economic impacts at country, regional and even global level, according to the Global Burden of Disease Study [28]. In our study, we did not find an association with HBV or HCV and CD4 changes or AIDS events and mortality. Most participants were from low‐middle income countries where uptakes of HCV treatment are recently being scaled up. In this cohort, 10% of PWID were co‐infected with chronic HBV. Currently, there are tolerable oral agents for HBV treatment (e.g. tenofovir); however, the accessibility of those drugs remains low in resource‐limited countries, especially where PWID have encountered barriers to engagement with the health system [29]. Therefore, the impact of chronic HCV and HBV infections on the comorbidities of HIV‐positive PWID remains unclear. Although our study did not include HCV treatment or liver‐related outcomes, future studies and evaluations of these outcomes are warranted to guide policy makers in the implementation of intervention strategies among PWID in the region. Nonetheless, regular, and adequate screening, and timely treatment of hepatitis infections are critical for PWID population.

Lower CD4 count was shown to be associated with new AIDS events or death in our study. Previous studies have suggested poorer treatment outcomes of PWID especially those with low CD4 count. Our study results are consistent with previous studies that demonstrated individuals with current CD4 counts less than 200 cells/µL having a higher risk for developing new AIDS events/death after ART initiation [30]. Our study also found that suboptimal drug adherence among PWID was associated with AIDS events or mortality.

We have evaluated TB as a separate outcome in this study. PWID tend to be at a greater risk of developing TB and are more likely to not be retained within the TB treatment programme [12]. TB incidence rate in the study was 1.01 (95% CI, 0.73 to 1.41) per 100 PYS in the cohort. This is remarkably higher than the general population of many Asian countries participating in the TAHOD cohort [31]. Our study found that prior TB diagnosis, lower current CD4 and suboptimal drug adherence were also associated with being diagnosed with a new TB event. This highlights the importance of routine screening of TB infections and latent TB therapy among PWID, especially among those with a history of TB and low CD4 in HIV clinics.

Like other non‐PWID participants in the cohort [20, 32], the majority of PWID participants presented late with a high prevalence of previous AIDS diagnosis. Studies have shown that PWIDs face different forms of barriers to treatment and healthcare services due to stigmatization and criminalization [33, 34, 35], which could largely delay HIV diagnosis or treatment initiation. Healthcare avoidance or HIV testing avoidance behaviours among PWID were also previously reported in the region [36, 37, 38]. Therefore, increasing awareness of the benefits of early HIV treatment initiation together with other supportive measures such as treatment adherence support [39], opioid substitution therapy [40] and harm reduction intervention such as needle syringe programmes [41] are needed to improve treatment outcomes among the population.

This study has several limitations. First, participants were designated as PWID based on the presumptive HIV mode of transmission defined at their enrolment in the cohort, and no data were collected regarding ongoing drug use or utilization of methadone therapy among our study participants. Therefore, we could not evaluate the impact of current illicit drug use on HIV treatment outcomes. The majority of PWID included in the study started ART in 2005 to 2010, which potentially limits the generalizability of our findings to the “treatment for all” period when ART is recommended after HIV diagnosis regardless of CD4 threshold, and when less toxic regimens became available. Another limitation is that the study group consists of a small number of PWID aged older than 50 years and a small number who were initiated with a PI‐based regimen as first‐line ART combination. As such, the generalizability of our findings to the general PWID is limited. Finally, we were not able to account for unobserved confounders within our cohort.

## 5 CONCLUSIONS

There has been improvement in immunological and virological responses after ART initiation among PWID. Earlier treatment initiation and implementation of adherence support programmes could benefit PWID within the region. Also, in the Asia‐Pacific region, routine HBV and HCV screening and treatment for this marginalized population with easy access to healthcare services are also critical. Policies enhancing HIV management, adherence support and prevention services tailored for the needs of PWID, and harm reduction services should be more widely adopted to control the HIV transmission in this population group.

## COMPETING INTEREST

The authors declare no conflict of interest related to this work.

## AUTHORS’ CONTRIBUTIONS

W.M.H., A.J. and A.A. contributed to study’s conception and design. W.M.H. and A.J. analysed the data. W.M.H. wrote the first draft. A.J., N.A.M.S., J.Y.C., B.V.H., J.R. and A.A. interpreted the data and critically reviewed the manuscript. All the authors have substantially contributed to the study and have reviewed and approved the manuscript.

## Supporting information


**Table S1**. Incidence rate tuberculosis and its associated factors among PWID
**Table S2**. Factors associated with CD4 changes over 10 years after ART initiation using random effect models
