# Supplementary material for: HIV treatment outcomes among people who acquired HIV via injecting drug use in the Asia‐Pacific region: a longitudinal cohort study
Source: J Int AIDS Soc. 2021 May 21;24(5):e25736. doi: 10.1002/jia2.25736 (PMC8140190; doi:10.1002/jia2.25736)
Supplement: Supplementary file 1 — Table S1. Incidence rate tuberculosis and its associated factors among PWID Table S2. Factors associated with CD4 changes over 10 years after ART initiation using random effect models [file JIA2-24-e25736-s001.docx]

**Supplementary Table S1**. Incidence rate tuberculosis and its associated factors among PWID

|  |  |  |  | | Univariate | | | Multivariate | | |
| --- | --- | --- | --- | --- | --- | --- | --- | --- | --- | --- |
|  | **Number of PWID** | **Incidence rate (/100pys)** | | **95% CI** | **HR** | **95% CI** | **p-value** | **HR** | **95% CI** | **p-value** |
| Total | 622 | 1.01 | 0.73-1.41 | |  |  |  |  |  |  |
| Sex |  |  |  | |  |  |  |  |  |  |
| Male | 578 | 1.03 | 0.73-1.45 | | Ref |  |  |  |  |  |
| Female | 44 | 0.82 | 0.21-3.29 | | 0.71 | 0.17-3.03 | 0.645 |  |  |  |
| Age at ART initiation* |  |  |  | |  |  |  |  |  |  |
| ≤30 | ~ | 0.94 | 0.39-2.25 | | Ref |  |  |  |  |  |
| >30 | ~ | 1.03 | 0.72-1.47 | | 2.33 | 0.87-6.25 | 0.094 | 3.40 | 1.24-9.34 | **0.017** |
| ART regimen* |  |  |  | |  |  | 0.793 |  |  |  |
| No treatment | ~ | 4.28 | 0.60-30.36 | | 2.39 | 0.23-23.78 | 0.473 |  |  |  |
| NRTI+NNRTI | ~ | 1.05 | 0.74-1.49 | | Ref |  |  |  |  |  |
| NRTI+PI | ~ | 0.59 | 0.15-2.34 | | 0.61 | 0.14-2.66 | 0.512 |  |  |  |
| Others | ~ | 0 | NA | | NA | NA | NA |  |  |  |
| HBV coinfection |  |  |  | |  |  |  |  |  |  |
| Negative | 434 | 0.94 | 0.62-1.42 | | Ref |  |  |  |  |  |
| Positive | 64 | 0.79 | 0.25-2.45 | | 0.78 | 0.23-2.69 | 0.696 |  |  |  |
| Not tested | 124 | 1.43 | 0.75-2.76 | |  |  |  |  |  |  |
| HCV coinfection |  |  |  | |  |  |  |  |  |  |
| Negative | 81 | 0.88 | 0.33-2.35 | | Ref |  |  |  |  |  |
| Positive | 440 | 0.88 | 0.58-1.33 | | 1.21 | 0.39-3.67 | 0.742 |  |  |  |
| Not tested | 101 | 1.82 | 0.95-3.50 | |  |  |  |  |  |  |
| History of AIDS diagnosis |  |  |  | |  |  |  |  |  |  |
| No | 287 | 0.6 | 0.33-1.12 | | Ref |  |  | Ref |  |  |
| Yes | 335 | 1.39 | 0.94-2.05 | | 2.13 | 1.01-4.54 | **0.04** | 1.59 | 0.51-4.95 | 0.424 |
| History of tuberculosis |  |  |  | |  |  |  |  |  |  |
| No | 433 | 0.66 | 0.40-1.07 | | Ref |  |  | Ref |  |  |
| Yes | 209 | 1.86 | 1.19-2.92 | | 2.28 | 1.12-4.64 | **0.022** | 2.69 | 1.23-5.87 | **0.013** |
| Ever smoke |  |  |  | |  |  |  |  |  |  |
| No | 45 | 0.60 | 0.15-2.42 | | Ref |  |  |  |  |  |
| Yes | 420 | 0.88 | 0.59-1.32 | | 1.87 | 0.37-9.34 | 0.448 |  |  |  |
| Not reported | 157 | 2.21 | 1.15-4.25 | |  |  |  |  |  |  |
| Above moderate drinking |  |  |  | |  |  |  |  |  |  |
| No | 72 | 0.98 | 0.44-2.18 | | Ref |  |  |  |  |  |
| Yes | 250 | 0.78 | 0.46-1.35 | | 0.79 | 0.25-2.54 | 0.719 |  |  |  |
| Not reported | 300 | 1.35 | 0.83-2.21 | |  |  |  |  |  |  |
| BMI*, kg/m^2^ |  |  |  | |  |  |  |  |  |  |
| <25 | ~ | 1.07 | 0.74-1.55 | | Ref |  |  | Ref |  |  |
| >=25 | ~ | 0.44 | 0.11-1.74 | | 0.24 | 0.53-1.10 | 0.09 | 0.33 | 0.07-1.43 | 0.138 |
| Not reported | ~ | 1.35 | 0.56-3.24 | |  |  |  |  |  |  |
| Year of ART initiation |  |  |  | |  |  | 0.004 |  |  |  |
| <2005 | 97 | 1.75 | 0.97-3.16 | | Ref |  |  |  |  |  |
| 2005-2010 | 336 | 1.32 | 0.87-2.00 | | 0.83 | 0.27-2.56 | 0.753 |  |  |  |
| >2010 | 159 | 0.17 | 0.04-0.69 | | 0.09 | 0.02-0.55 | 0.009 |  |  |  |
| Adherence* |  |  |  | |  |  |  |  |  |  |
| <= 95% | ~ | 1 | 0.20-10.03 | | Ref |  |  | 24.23 | 1.62-42.19 | **0.013** |
| >95% | ~ | 0.18 | 0.51-1.45 | | 0.02 | 0.001-0.41 | **<0.001** | Ref |  |  |
| Not reported | ~ | 1.83 | 1.28-2.60 | |  |  |  |  |  |  |
| Income |  |  |  | |  |  |  |  |  |  |
| Low-middle income countries |  | 0.9 | 0.61-1.34 | | 0.9 | 0.57-1.43 | 0.653 |  |  |  |
| High income countries |  | 1.45 | 0.78-2.69 | | Ref |  |  |  |  |  |
| CD4+ cell count* (cells/mm^3^) |  |  |  | |  |  | **0.002** |  |  | **0.015** |
| ≤50 | ~ | 2.30 | 1.38-3.81 | | 8.92 | 3.25-24.45 | **<0.001** | 5.53 | 2.05-14.97 | **0.001** |
| 51-100 | ~ | 1.26 | 0.70-2.28 | | 2.94 | 0.79-11.01 | 0.109 | 2.15 | 0.56-8.35 | 0.267 |
| 101-200 | ~ | 0.25 | 0.06-1.00 | | 1.05 | 0.29-3.77 | 0.944 | 0.80 | 0.22-2.89 | 0.737 |
| >200 | ~ | 0.32 | 0.10-0.99 | | Ref |  |  | Ref |  |  |
| Not done | ~ | 2.17 | 0.81-5.78 | |  |  |  |  |  |  |
| HIV viral load* (copies/mL) |  |  |  | |  |  | **0.02** |  |  | 0.336 |
| <=400 | ~ | 0.40 | 0.19-0.83 | | Ref |  |  | Ref |  |  |
| 401-100000 | ~ | 0.56 | 0.14-2.23 | | 1.04 | 0.21-5.16 | 0.960 | 0.50 | 0.11-2.72 | 0.422 |
| >100000 | ~ | 4.33 | 1.80-10.40 | | 5.95 | 1.64-21.59 | **0.007** | 1.74 | 0.37-8.32 | 0.485 |
| Not done | ~ | 1.73 | 1.13-2.65 | |  |  |  |  |  |  |

**Supplementary Table S2**. Factors associated with CD4 changes over 10 years after ART initiation using random effect models

|  | Univariable | | | Multivariable | | |
| --- | --- | --- | --- | --- | --- | --- |
| Total 435 PWID participants | **Difference** | **95% CI** | **p-value** | **Difference** | **95% CI** | **p-value** |
| Time-varying age (years)* |  |  |  |  |  |  |
| ≤30 | Ref |  |  | Ref |  |  |
| >30 | -8.5 | (-28.9, 12.1) | 0.417 | -7.6 | (-27.9, 12.6) | 0.461 |
| Sex |  |  |  |  |  |  |
| Male | Ref |  |  |  |  |  |
| Female | -11.5 | (-74.9, 51.9) | 0.723 |  |  |  |
| Year of ART initiation |  |  | 0.168 |  |  |  |
| <2005 | Ref |  |  |  |  |  |
| 2005-2010 | 31.1 | (-22.8, 84.9) | 0.259 |  |  |  |
| >2010 | -18.7 | (-77.6, 40.3) | 0.535 |  |  |  |
| HIV viral load (copies/mL)* |  |  |  |  |  | **<0.001** |
| ≤ 400 | Ref |  |  | Ref |  |  |
| 401-100,000 | -92.5 | (-129.6, -55.6) | <0.001 | **-65.5** | **(-95.6, -35.3)** | **<0.001** |
| >100,000 | -157.7 | (-223.5, -92.3) | <0.001 | **-121.9** | **(-175.4, -68.4)** | **<0.001** |
| Not reported |  |  |  |  |  |  |
| Pre-ART CD4 (cells/uL) |  |  | <0.001 |  |  | **<0.001** |
| ≤ 50 | Ref |  |  | Ref |  |  |
| 51-100 | -46.5 | (-88.0, -5.1) | 0.028 | **-45.2** | **(-84.2, -6.3)** | **0.023** |
| 101-200 | -60.0 | (-98.4, -21.5) | 0.002 | **-69.4** | **(-105.6, -33.2)** | **<0.001** |
| > 200 | -89.5 | (-132.5, -46.5) | <0.001 | **-96.9** | **(-137.2, -56.4)** | **<0.001** |
| BMI (kg/m^2^)* |  |  | 0.111 |  |  |  |
| < 25 |  |  |  |  |  |  |
| ≥ 25 | 33.5 | (-11.8, 66.1) | 0.157 |  |  |  |
| Not reported |  |  |  |  |  |  |
| Adherence* |  |  |  |  |  |  |
| ≤ 95% | Ref |  |  |  |  |  |
| > 95% | 105.3 | (53.9, 157.5) | <0.001 |  |  |  |
| Not reported |  |  |  |  |  |  |
| ART regimen* |  |  |  |  |  |  |
| No treatment | -11.2 | (-36.4, 188.5) | 0.592 |  |  |  |
| NRTI+NNRTI | Ref |  |  |  |  |  |
| NRTI+PI | 81.5 | (-18.5, 187.9) | 0.165 |  |  |  |
| Others | 22.5 | (-88.4, 199.2) | 0.312 |  |  |  |
| Ever smoke |  |  |  |  |  |  |
| No | Ref |  |  |  |  |  |
| Yes | 5.5 | (-51.2, 62.1) | 0.850 |  |  |  |
| Not reported |  |  |  |  |  |  |
| Hepatitis B co-infection |  |  |  |  |  |  |
| Negative | Ref |  |  |  |  |  |
| Positive | -17.5 | (-67.4, 32.3) | 0.491 |  |  |  |
| Not tested |  |  |  |  |  |  |
| Hepatitis C co-infection |  |  |  |  |  |  |
| Negative | Ref |  |  |  |  |  |
| Positive | -33.1 | (-80.3, 14.0) | 0.169 |  |  |  |
| Not tested |  |  |  |  |  |  |
| History of AIDS Diagnosis |  |  |  |  |  |  |
| No | Ref |  |  |  |  |  |
| Yes | 8.2 | (-22.9-39.4) | 0.604 |  |  |  |
| History of tuberculosis |  |  |  |  |  |  |
| No | Ref |  |  |  |  |  |
| Yes | 4.3 | (-30.9, 39.5) | 0.811 |  |  |  |
| All model covariates were stratified by site. Global p-values for age and CD4 are tests for trend. All other global p-values are tests for heterogeneity excluding missing values. Time from ART initiation was also adjusted in the final model.  *Age, HIV viral load, BMI, ART regimen and adherence data are time-varying variables.  P-values in bold represent significant covariates in the final model.  Abbreviations: ART, antiretroviral therapy; NRTI, nucleotide reverse transcriptase inhibitor; NNRTI, non-nucleotide reverse transcriptase inhibitor; PI, protease inhibitor; BMI, body mass index. | | | | | | |
